# Worldwide Trend Observation and Analysis of Sheep Pox and Goat Pox Disease: A Descriptive 18-Year Study

**DOI:** 10.3390/v17040479

**Published:** 2025-03-27

**Authors:** Juana Bianchini, Maria-Eleni Filippitzi, Claude Saegerman

**Affiliations:** 1 Research Unit of Epidemiology and Risk Analysis Applied to Veterinary Science (UREAR-ULiège), Fundamental and Applied Research for Animals & Health (FARAH) Center, Faculty of Veterinary Medicine, University of Liège, 4000 Liege, Belgium; juana.bianchini@uliege.be; 2 Laboratory of Animal Health Economics, School of Veterinary Medicine, Faculty of Health Sciences, Aristotle University of Thessaloniki, 54124 Thessaloniki, Greece; mefilippi@vet.auth.gr

**Keywords:** sheep and goat pox, gross domestic product per capita, endemic status, change-point analysis, trends observation, trends analysis

## Abstract

Sheep and goat pox (SGP) are animal diseases of important economic impact which have been emerging into new geographic areas, including occasional incursions in disease free countries. The main objective of this study is to observe and analyse the global distribution of SGP during an 18-year period (2005–2022). Countries’ SGP epidemiology was characterised by classifying them according to the frequency of reporting years. A negative binomial regression model was used to test for associations between the economic status of a country, the sheep and goat populations, the continent, and the likelihood of an SGP outbreak occurring. A change-point analysis was used to determine significant change points of outbreaks for 18 years. Countries which presented high endemic status were mostly located in the North African region, the Middle East, and Asia, in particular India and China. Economic status was found to be significant for outbreak occurrence in endemic countries, in contrast to countries with outbreaks occurring where other socio-economic factors influence the disease occurrence. The total sheep and goat population was found to be significantly associated with countries and regions. The change-point analysis showed that changes in outbreak occurrence were observed when countries with most reported outbreaks controlled the diseases. While the husbandry and social conditions that exist in certain regions, particularly of Africa and Asia, make the prospect of SGP eradication highly unlikely, an effective implementation of vaccination strategies and control policies would decrease the incidence of SGP, improving animal health and economics in affected countries.

## 1. Introduction

In the livestock industry, sheep and goats are one of the primary animal species as they contribute significantly to the world economy. Sheep pox and goat pox are two highly infectious viral diseases of ovines and caprines which have a substantial impact on these populations. The causative agents of these diseases are sheeppox virus (SPPV) and goatpox virus (GTPV) that, along with lumpy skin disease virus (LSDV) which affects bovines, belong to the *Capripoxvirus* genus of the *Poxviridae* family [1]. Most examined strains of SPPV and GTPV cause more severe clinical disease in either sheep or goats, while some strains that have been isolated appear to be equally pathogenic in both species [2]. Their main mode of transmission occurs through aerosol and direct contact between animals, with mechanical transmission by insect vectors playing a minor role [3]. The virus, however, can survive in the environment, and thus indirect transmission may also occur through fomites such as human movements, vehicles, and trade of hides when insufficiently treated [4]. Wildlife is assumed to not play a relevant role in the epidemiology of sheep pox (SPP) and goat pox (GTP), although it cannot be excluded as wild sheep and wild goats can be infected [2]. The diagnosis can be made under field conditions, with the clinical diagnosis performed by trained veterinary staff which is effective for the early detection of outbreaks. The laboratory diagnostics tests considered the most sensitive and specific are polymerase chain reaction (PCR) assays and enzyme-linked immunosorbent assays (ELISAs), for the detection the nucleic acid of the virus and antibodies against it, respectively [4].

Affected animals present reduced milk production, decreased weight gain, abortions, poor quality of wool and hides, increased susceptibility to other infectious diseases, and increased morbidity and mortality [2,5]. The aforementioned consequences of these diseases on animal health, together with the additional trade restrictions resulting from animals being affected and the cost of control/eradication measures (e.g., stamping out, disease surveillance, movement control) inevitably lead to considerable economic losses [6].

Besides the important economic impact of these diseases at the national level, from a productivity point of view, in endemic countries, they reduce the productive potential of the existing sheep and goat industries, they limit the development of intensive feed-lot systems, and they hamper the improvement of indigenous breeds of sheep and goats with exotic breeds [3,7,8,9]. Therefore, their impact is substantial to the livelihoods of farmers, especially small-scale farmers in poor, rural communities. Regarding the eradication and control-associated costs at a farm level, these can be high and may remain for a long time [10]. For example, it was calculated that it took 6 years for a flock in India to recover from an outbreak in which the mortality rate had been 49.5% [5].

Given their economic importance as described, the World Organisation for Animal Health (WOAH) has categorised sheep pox and goat pox as notifiable diseases with compulsory notification to the WOAH for the EU Member States and its trading partners [1]. WOAH consider sheep pox and goat pox to be a single disease entity, and here onwards it will be referred to as sheep and goat pox (SGP).

The incidence of SGP has decreased in the last 50 years and it has been eradicated from many developed countries [11]. For instance, it was eradicated from Britain in 1866, and from France, Spain, and Portugal in 1967, 1968, and 1969, respectively. However, sporadic outbreaks still occur in some European countries, e.g., in Bulgaria in 2013, in Greece in the years 2006–2007, and in 2024 and in Spain in 2022 and 2023 [12]. In contrast to European countries (in EU vaccination has implications in trade and product prices, e.g., in Greece, the authorities do not want to vaccinate to align with EU criteria for freedom status and to ensure trade conditions in the long run), in many non-European countries, SGP remains endemic despite the availability of vaccines (i.e., a live vaccine inducing long protection and an inactivated one inducing short protection). These are countries in the North and Central African continent, the Middle East, Turkey, and some parts of Asia. Over the last decade, SGP has also expanded its incidence, being reported in new countries further into Southeast Asia, such as Vietnam and Indonesia [12].

Due to its economic importance and the recurrence and expansion of SGP into new countries, the aim of this study was to describe (i.e., trends observation) and analyse (i.e., trends analysis) changes in SGP-affected countries during an 18-year period (2005–2022). The specific objectives of this study were to (i) characterise the countries’ SGP epidemiology over the last 18 years (2005–2022) according to their SGP reporting, (ii) determine if the economic status of a country, sheep and goat animal populations, and continent have an effect on the presence and occurrence of outbreaks of the disease, and (iii) present the geographical evolution of SGP outbreaks from 2005 to 2022.

## 2. Materials and Methods

### 2.1. Data and Variables

SGP outbreak data were obtained between 1 January 2005 and 31 December 2022 from the World Animal Health Information System (WAHIS) [12], the global animal health reference database of the WOAH. These data are submitted to the WOAH by the National Veterinary Authorities of Member countries of the institution in semester reports. The reports present information on the number of outbreaks and cases per year, semester, world region, country, administrative division, animal species affected, number of deaths, and of vaccinated animals. For the purpose of the present study, the data extracted were the number of outbreaks and cases, the year, the world region, country, and administrative region. Countries which reported the disease were included in the dataset for the entire study period. Outbreak data were aggregated by semester and reported in years.

Data of the sheep and goat animal population for each year of the study were extracted from the Food and Agriculture Organization of the United Nations (FAO) database [13]. The countries for which data were extracted were those with at least one outbreak reported during the study period. There were no records on the animal population for the year 2022, and thus a simple linear regression using the available last three years of recorded animal population of a country was used to estimate the number of sheep and goats per country for the year 2022. The total number of sheep and goats were additionally categorised in four groups according to their quartiles: low number, low middle, high middle, and high number.

Information on national Gross Domestic Product (GDP) per capita in US dollars deflated to the year 2015 for each country per year and their classification according to their income (low-income country, lower-middle income, upper-middle income, and high-income country) was extracted from the World Bank and included in the database [14].

All three datasets were checked for missing values and inconsistencies, following which they were collated into a single dataset for the analysis.

### 2.2. Data Processing

#### 2.2.1. Country Classification Based on Their SGP Reporting

To classify the SGP status of a country, the two WAHIS semester reports by country were aggregated yearly. Subsequently, to estimate the frequency of number of years positive during the study period, the total number of years with SGP presence (years reported) of a given country was divided by the number of years with SGP reporting by country to WAHIS. This frequency, expressed in percentage obtained, was used to classify the countries into four categories from 2005 to 2022: (a) highly endemic (>70%); (b) endemic (31–69%); (c) low endemic (11–30%), and (d) with sporadic outbreaks (≤10%). The countries were then mapped according to this obtained frequency using Quantum GIS version 3.34.1 (Quantum GIS Development Team, 2023).

#### 2.2.2. Region Classification

The WAHIS database has five world categories of countries based on continents. For better display of the data (fit to purpose), these established categories were further subdivided into regions. Countries of the African continent were categorised into two regions: (a) North Africa, including countries bordering the Mediterranean Sea; (b) and the Sub-Saharan region, which included the remaining countries of the African continent. Countries in the Asian continent were classified into the following three regions: (a) Middle East, which included countries in the Arabian Peninsula; (b) Central Asia, which included countries bordering the Arabian Peninsula, countries in the Caucasus region (excluding Russia), Turkey, and Iraq; and (c) East Asia, which included the countries bordering the Indian Ocean, China, and neighbouring nations. No categorization was made for European countries as preliminary analysis showed only four countries in this category (i.e., Spain, Bulgaria, Greece, and Russia). Russia, although geographically situated in Asia, was classified as Europe, in accordance with the WAHIS database.

#### 2.2.3. Data Validation

Outbreak data extracted from the WAHIS database were checked to identify if zero outbreak data was due to an unavailable report (i.e., no report was submitted from a specific country in a year) or if zero outbreaks were reported for the particular year. If there was no report submitted, outbreaks were recorded in the database as missing value. For reports with zero reported outbreaks but with registered cases, the total number of outbreaks was calculated according to the administrative region level where the cases were reported from (i.e., the total number of cases reported was divided by the total number of administrative regions where the cases were reported from). If no information on where these cases occurred was available, a minimum of one outbreak was considered per country.

### 2.3. Statistical Analysis

For the comparison of the GDP and population size with the four SGP status classification of countries and to compare differences between the different categories of regions and the sheep and goat populations, univariate analytical methods were used.

The non-parametric Kruskal–Wallis test was used to evaluate if there were differences between the median GDP per capita and the sheep and goat population size between the four SGP status classification categories of countries (i.e., highly endemic, endemic, low endemic and with sporadic outbreaks). A post hoc Mann–Whitney U test using a Bonferroni-adjusted *p*-value (*p*-value 0.05/number of pairwise comparisons) was used for pairwise comparison. Additionally, the sheep and goat population in the six classified regions (i.e., Middle East, Sub-Saharan Africa, North Africa, Central Asia, East Asia, and Europe) were compared using the same aforementioned non-parametric tests.

The relationship between the SGP status of the countries and (a) the category of number of sheep and goats (i.e., low number, low middle, high middle, and high) and (b) their classified economic status level of income was evaluated using univariate logistic regression models. A binary outcome variable was then generated and was named as ‘present’ (yes–no). This outcome variable that took a value of one for each country which was classified as highly endemic, endemic, or low endemic and took a value of zero in the case of a country classified as having sporadic outbreaks.

To establish if the level of income category and the categories of sheep and goat population were associated to presenting an outbreak after preliminary descriptive analysis, it appeared that the data suggested overdispersion (i.e., data variance greater than the mean). Consequently, a negative binomial regression model was used to model the association between the outcome variable, outbreak occurrence per country, and explanatory variables of interest: category of number of sheep, category of number of goats, level of income of the country and region. Univariate regressions were performed for each variable and the outcome variable and were included in the multivariable model at *p*-value < 0.20. Variables were retained in the final multivariable model at *p*-value < 0.05. The negative binomial coefficients were reported as the incident rate ratio (IRR) form.

### 2.4. Trends Analysis

To determine significant changes in the number of SGP outbreaks over time, descriptive graphical methods were firstly used and then change-point analysis was applied to the total yearly outbreak data (i.e., not subdivided by continent nor region) to identify significant changes in the number of SGP outbreaks over the 18-year period. A likelihood-based change point detection approach was utilised to detect changes in the mean and variance of the number of SGP outbreaks [15].

Records were managed using Microsoft Excel^®^ 2019 (Microsoft Excel for Windows; Microsoft Corporation, Redmont, WA, USA). Statistical analyses were performed using STATA SE.14.2^®^ (College Station, TX, USA) and R^®^ (R version 4.3.2).

## 3. Results

### 3.1. Description of Countries with the Presence of SGP

Between January 2005 to December 2022, there were a total of 67 countries which reported presence of SGP in a given year (Table 1). Africa had a total of 30/67 countries reporting presence SGP (*n* = 5 in the North Africa region and *n* = 25 in the Sub-Saharan region); Asia, a total of 33/67 countries, with the Middle East and East Asia regions having 12/67 countries each and Central Asia with 9 countries. Europe had four countries reporting SGP.

**Figure 1:**
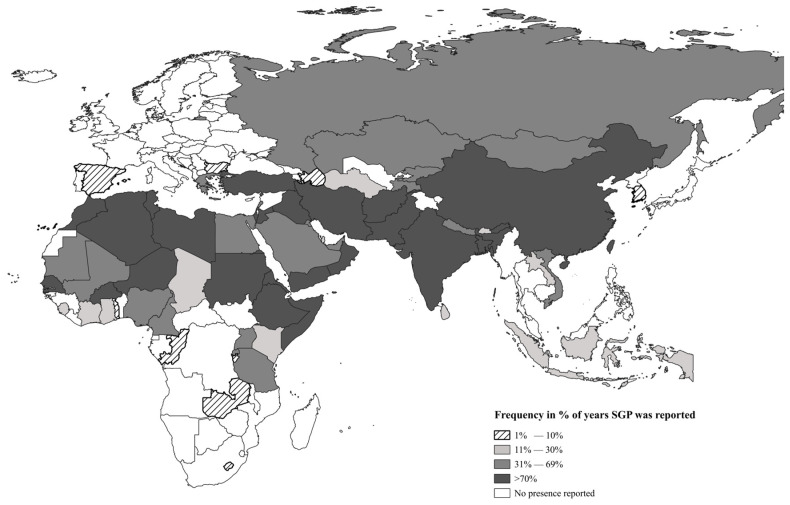
Frequency of years of presence of SGP in different countries which reported to the WOAH for the period 2005–2022. Total number of countries *n =* 67.

Figure 1 shows the global occurrence of SGP from 2005 to 2022 in percentage (i.e., number of years positive during the study period divided by the number of years with SGP reporting, expressed in percentage). In relation to the frequency of years of reporting the presence of SGP and their corresponding classification, from the 67 countries, 24 were classified as highly endemic, 21 as endemic, 13 as low endemic, and 9 as having sporadic outbreaks. Of the total of the countries classified as highly endemic and endemic (*n* = 45), in decreasing order, 31.1% belonged to the Sub-Saharan region, 24.4% to the Middle East, 15.56% to the central Asian region, 13.3% to the East Asian region, 11.1% to the North African region, and 4.4% to Europe. Of the 67 countries which reported the presence of SGP, 14 were excluded from the statistical analysis on the basis that 9 countries (Bangladesh, Indonesia, Laos, Lebanon, Qatar, Rwanda, Saudi Arabia, Togo, and Zambia) only recorded the presence or suspicion of SGP, with no records of quantitative data, 3 (Burundi, Sierra Leone, Gambia) had less than 50% of number of reports for the whole study period, and 2 (Palestine and Taiwan) had no GDP data.

### 3.2. Level of Income, Sheep and Goat Population, and Countries’ SGP Status

**Figure 2:**
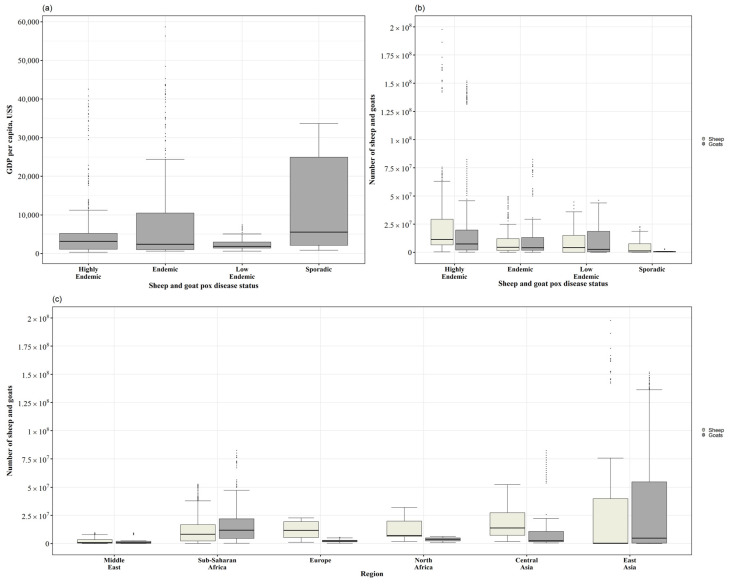
Box plots for the different categories. (**a**,**b**) The sheep and goat pox disease status by GDP per capita in USD and (**c**) the number of sheep and goat population by classified region. Total number of countries = 53.

Countries classified as having sporadic outbreaks had the highest median GDP per capita, followed by highly endemic countries (Figure 2a), with statistical difference in their GDP median (Kruskal–Wallis; *p*-value < 0.05). The post hoc analysis showed that there was no statistically significant difference between the median GDP per capita of the groups ‘highly endemic’, ‘endemic’, and ‘low endemic’. The difference was only identified between ‘sporadic outbreaks’ countries and the other three status categories (Mann–Whitney U test; Bonferroni-adjusted *p*-value < 0.008). The univariate logistic regression showed that the odds of being classified as ‘present’ (i.e., not sporadic) were similar between the different income categories, ranging from OR 0.26 to 0.3 (Table 2). The higher the income category, the less likely the country was to ‘present’ SGP.

The number of sheep and goat population according to the country’s epidemiological situation is shown in Figure 2b. There was a difference in the median between the groups of SGP status (Kruskal–Wallis *p*-value < 0.05). The post hoc analysis test (Mann–Whitney U test; Bonferroni-adjusted *p*-value < 0.008) showed that the median population number of both sheep and goats were different between the SGP status of the countries, except for endemic and low endemic countries, i.e., these two groups had no statistical difference in sheep and goat populations. The results of the univariate logistic regression showed that the odds of having SGP ‘present’ (i.e., not sporadic) were highest in the category of high number of sheep, OR 7.66, whilst for goats, the middle high category had the highest, OR 13.97 (Table 2).

There were statistical differences in the total number of sheep and goat population between the classified regions (Mann–Whitney U test *p*-value < 0.05) (Figure 2c). The post analysis of the rank sum test showed there was no significant difference (Bonferroni-adjusted *p*-value > 0.003) between the number of sheep population among North Africa, Sub-Saharan Africa, and the Middle East. Europe had statistical differences with all the regions except for Central Asia, whilst Central Asia, besides Europe, had no statistical difference with North Africa. East Asia had no statistical difference between Middle East and North Africa (Table A2).

The top three countries with the highest median number of sheep by region were, for East Asia, China (median = 152,773,500); India (median = 69,228,295); and Mongolia (median = 21,640,604). For Central Asia they were Iran (median = 46,399,505); Pakistan (median = 28,925,000); Turkey (median = 28,354,740). In the Middle East region, Yemen, Iraq, and Jordan were the top three, with total median number of 9,915,718, 6,678,751, and 2,544,880, respectively. In the North Africa region, the top three were Algeria (median = 27,200,000); Morocco (median = 18,992,892); and Libya (median = 7,150,000). In the Sub-Saharan region, they were Sudan (median = 40,978,159); Nigeria (median = 40,801,415); and Ethiopia (median 28,120,156). The sheep population for Europe’s four countries were Bulgaria (median = 1,364,766); Greece (median = 8,785,094); Russia (median = 20,252,310); and Spain (median = 16,228,980).

The number of goats per region shows that East Asia, followed by the Sub-Saharan African region, had the highest number of goats (Figure 2c). The post analysis when comparing between groups of regions showed that there was a statistical difference between most the regions (Bonferroni-adjusted *p*-value < 0.003). The number of goats was not significantly different between Central Asia, East Asia, and North Africa. The other two regions with no statistical difference were East Asia and Sub-Saharan Africa (Table A3).

The top three countries with highest median number of goats per region were for China (median = 138,780,343); India (median = 138,930,504); and Mongolia (median = 20,989,148) for the East Asian region. In the Central Asian region, they were Iran (median = 20,499,300); Pakistan (median = 65,736,500); and Turkey (median = 8,791,417). In the Middle East region, they were Yemen (median = 9,093,959); Oman (median = 2,106,058); and the United Arab Emirates (median = 2,061,495). For the North Africa Region, they were Morocco (median = 5,708,350); Algeria (median = 4,908,327); and Egypt (median = 4,092,748). In the Sub-Saharan region, they were Nigeria (median = 71,328,715); Sudan (median = 32,130,097); and Ethiopia (median *n =* 28,638,151). In Europe, Bulgaria (median = 293,142); Greece (median = 4,838,624); Russia (median = 2,097,805); and Spain (median = 2,828,182) formed the top four countries.

### 3.3. Outbreak Trend

Descriptive information on the outbreak data is presented in Table 3. The 18-year study period had a total of 37,606 outbreaks represented by 53 countries. The lower-middle-income category had the highest number of outbreaks (*n* = 19,394) represented by the highest number of countries 25/53 (47.17%). The high-income category had the lowest number of total outbreaks (*n =* 4748 outbreaks coming from 8 countries). Additionally, it also had the lowest median number of sheep and goats. The upper-middle-income category had the second lowest number of outbreaks (*n* = 5825), with the highest number of sheep population (median = 13,800,000 sheep), whilst the highest number of goats (median = 13,800,000) was found in the low-income category.

In the multivariable model, the level of income was not found to be significantly associated with the risk of outbreak (*p*-value > 0.25), whereas the other three variables (i.e., category of number of sheep, category of number of goats, income category and region) were significant. In the multivariable model, the number of sheep and goats were associated with the incidence risk of an outbreak. The high category had the highest incidence risk ratio for sheep (IRR = 9.87 with 95% CI: 3.89–25.03) and goats (IRR = 13.85 with 95% CI: 5.43–35.29) compared to the low category (Table 4). Regarding the geographical region, the Middle East (IRR = 76.63 with 95% CI: 30.74–191.01) and North Africa had a higher likelihood of having an outbreak (IRR = 36.14 with 95% CI: 15.02–86.95) compared to Europe.

**Figure 3:**
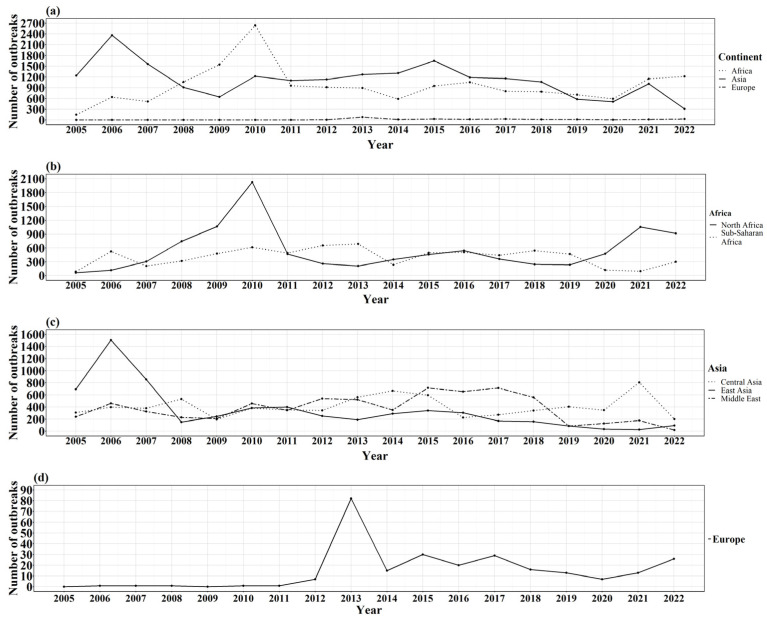
Line plots of SGP outbreaks per year from 2005 to 2022. (**a**) SGP outbreaks by continent. (**b**) SGP outbreaks by region of Africa. (**c**) SGP outbreaks by region of Asia. (**d**) SGP outbreaks of Europe.

The outbreak trend through the 18-year period showed that the year 2010 had reported the highest number of outbreaks (*n =* 3864), whereas the year with the least number of outbreaks was 2020 (*n =* 1111) (Figure 3a). Africa had a total of 17,145 outbreaks, representing 45.59% of reported outbreaks, followed by Asia (*n =* 20,198 representing 53.71%) and Europe (*n* = 263 representing 0.70%).

Africa (*n =* 24 countries) had a steady increase of outbreaks per year, reaching its peak number of outbreaks in 2010 (*n* = 2641), with the following year, 2011, presenting a sharp decrease (*n =* 956). In the last two years of the study period, an increase occurred, with the year 2021 registering 1149 outbreaks and 2022 registering 1215 outbreaks. When observing the two African regions, the North African region (*n* = 5 countries) reported the highest number of outbreaks (*n =* 9889), with the Sub-Saharan region (*n* = 19 countries) having 7256 outbreaks. The North African region showed sharp increases and decreases during the 18 years whilst the Sub-Saharan region had a more undulating pattern (Figure 3b).

Morocco, Ethiopia, Tunisia, Niger, Algeria, and Somalia were the countries with the highest total number of outbreaks during the whole study period. In detail, these were as follows: Morocco (*n =* 4766 representing 27.80%), Ethiopia (*n* = 3371 representing 19.66%), Tunisia (*n* = 3329 representing 19.42%), Niger (*n* = 2532 representing 14.77%), Algeria (*n* = 1720 representing 10.03%), and Somalia (*n =* 568 representing 3.31%).

**Figure 4:**
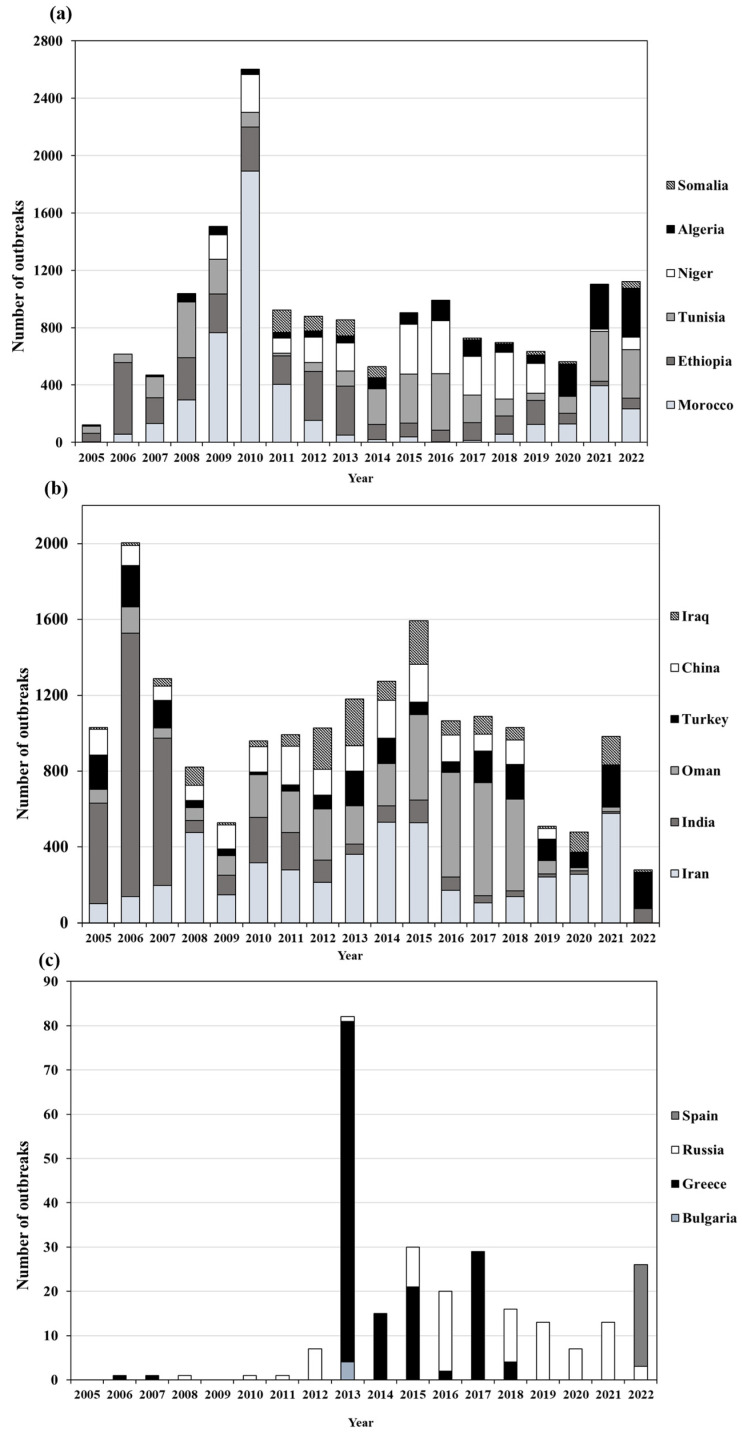
Top 6 countries in number of outbreaks for (**a**) Africa (**b**) Asia and (**c**) the four countries in Europe that reported outbreaks.

Morocco accounted for the highest number of outbreaks (*n* = 1890) in the year 2010. The country in the first two years of the study period (2005 and 2006) had a low outbreak number (*n* = 5 and *n* = 57 respectively) and from 2013 to 2018, outbreaks were kept to a number below 100. After this period, it increased and reached 395 outbreaks in 2021 and 234 outbreaks in 2022. Ethiopia was the country with most outbreaks from 2005 to 2007 and 2012 to 2013. From 2008 to 2011 it stayed in second place for both 2018 and 2019. Tunisia reported four years with outbreaks above 300: in 2008 (*n =* 386), 2016 (*n* = 394), 2021 (*n =* 348), and 2022 (*n* = 341). Algeria reported zero outbreaks in 2006 and in the last three years of the study (2020–2022) it reported the highest number of outbreaks (*n* = 227, *n* = 309, *n* = 340, respectively). Somalia, during the first six years of the study period (2005–2010), had no outbreaks reported, with 2011 making the year with its highest reported number outbreaks *n* = 155, steadily decreasing its outbreaks after that year (Figure 4a). Niger, in the years 2012, 2013, and 2016, was the country in second place for reported outbreaks and between the years 2017 and 2019, it was the country with most outbreaks.

Regarding the Asian continent (*n =* 25 countries), the regions with most outbreaks were Central Asia (*n =* 9 countries), with a total of 7203 outbreaks, followed by Middle East (*n* = 8 countries), *n =* 6495 outbreaks, and East Asia (*n* = 8 countries), with *n =* 6178 outbreaks. The year in which Asia had the most recorded outbreaks was 2006 (*n =* 2364). The first four years of the study period (2005–2009) had the greatest number of countries reporting the disease (*n* = 17 outbreaks) and 2021 and 2022 had the least (*n =* 9 outbreaks) (Figure 3).

The six countries that presented the highest percentage of outbreaks in Asia were Iran, India, Oman, Turkey, China, and Iraq representing 23.65%, 19.50%, 18.63%, 10.58%, 9.65%, and 7.78% of the total outbreaks, respectively. Iran reported the highest total number outbreaks (*n =* 4777), with the year 2021 having the maximum of outbreaks (*n* = 578). Additionally, Iran was the country with most reported number of outbreaks for 10 of the years of the study period (2008–2011, 2013–2015, 2019–2021). India was the country with highest reported outbreak in the first three years of the study period in 2005 (*n* = 529), in 2006 (*n =* 1389), and in 2007 (*n =* 777). Oman showed a gradual increase in outbreaks and between 2016 and 2018 it was the country with the greatest number of outbreaks (2016 (*n* = 551), 2017 (*n* = 596), and 2018 (*n* = 484)). Turkey reported outbreaks throughout the whole study period in an undulating pattern. The peaks with the most outbreaks recorded were in the years 2006 (*n* = 219), 2013 (*n* = 182), 2018 (*n* = 183), and 2021 (*n* = 224). China reported its lowest number of outbreaks in 2019 (*n* = 56), with the years 2014 and 2015 having 200 outbreaks each (Figure 4b). Iraq was always in the top six reporting countries but was never the highest reporting country in any given year. 

Four countries in the European region reported SGP during the 18-year study period. No outbreaks were recorded in the first year 2005 or 2009. The peak number of outbreaks (*n* = 82) was reported in 2013, with Greece as main contributor (*n* = 77) (Figure 3d). During this year, Bulgaria (*n* = 4) and Russia (*n* = 1) also reported some outbreaks. The rise observed in the last year of the study period 2022 is largely due to Spain, with 23 outbreaks, followed by Russia with 3 outbreaks (Figure 4c).

**Figure 5:**
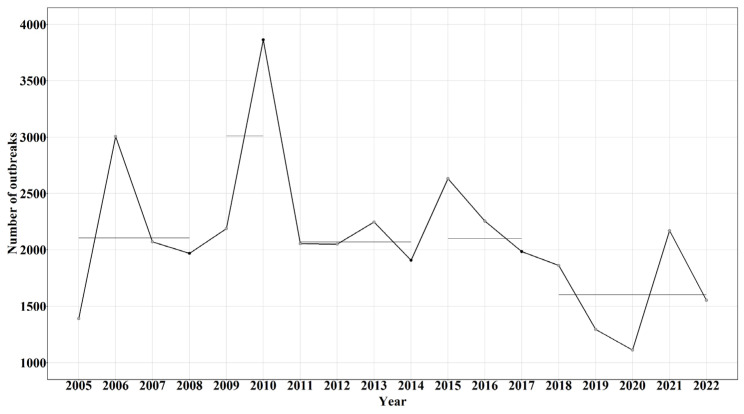
Change points in time series of SGP outbreak reports between the years 2005 and 2022. Black dots are change points and straight lines are the corresponding segments.

The SGP outbreak time-series data from 2005 to 2022 for SGP has four change points and five segments corresponding to them (Figure 5). In the present study, each segment represents the mean of the number of SGP outbreak reports submitted during the period corresponding to that segment. After the first detected change point, it is observed that the second segment had the highest mean number of SGP outbreaks reported, which corresponds to the years 2009 and 2010. The first segment, from 2005 to 2009; the third segment, from 2011 to 2014; and the fourth segment, 2015 to 2017, had a similar mean number of SGP outbreaks. The fourth segment detected a change in 2018, with the lowest mean number of SGP outbreaks reported in the whole study period.

## 4. Discussion

The data presented here show that although SGP is a notifiable disease to the WOAH it is a neglected disease, which is not taken into account until there is a major outbreak or it presents itself in a new territory. This is reflected in the fact that there is an absence of SGP reports submitted to the WOAH, particularly in the last three years of the study period (2020 to 2022) and there is lack of quantitative data from other countries, i.e., they report the presence of the disease but do not report any quantitative data (e.g., Saudi Arabia, Indonesia).

When observing the geographical reporting of SGP presence in countries in the 18-year study period, it is noted that overall, the disease has been present in the same countries previously recorded; it is still endemic in North and Central African countries, the Arabian Peninsula, Central Asia, and East Asia. This is consistent with earlier reports of SGP [2,4,6], which shows that its distribution has been relatively stable in the last 10 years. It has, however, expanded towards Southeast Asia, with a reported presence in Vietnam, Laos, and Indonesia. The single African country situated in the South of the continent which reported the disease was Lesotho. Interestingly, this particular country is not geographically situated where the disease is endemic, nor does it have any neighbouring countries which register outbreaks. No information on how the disease entered Lesotho is available, but as SGP’s most common form of entry into a country is through the legal/illegal animal trade [4], it most likely it happened through the introduction of an undetected infected animal.

The descriptive results of the study show that two thirds (75%) of the countries reporting SGP were classified as highly endemic and endemic, of which 85% were developing countries categorised as low income and lower middle income. The economic resources of a country have an effect on controlling livestock infectious diseases. Certainly, with higher economic power, there are more resources for better diagnosis, surveillance, and control or eradication. However, the results obtained here show that although low-income countries represent the highest endemic level, this was not strongly associated with their economic status (in GDP). There was no statistically significant difference between the median GDP per capita of countries where the disease was present for more than 10% of the years of the study period (i.e., highly endemic, endemic, and low endemic); only countries classified as sporadic had a statistical difference in the median GDP with the other categories. This is also observed when measuring the association between sporadic countries and those where the disease was present in the univariate analysis. Indeed, the odds ratio of the income categories, lower middle income, upper middle income and high income had similar odds ratio when compared to the reference category low-income.

This could imply that the economic resources of a country is not the only factor which influences the occurrence of SGP and that other regional characteristics may be in play. Indeed, there were six countries classified here as highly endemic or endemic that are in the high-income category, almost all belonged to the Middle East region. This region has oil-rich countries which have the economic resources that could enable the implementation of control measures and vaccination to reduce the disease outbreak numbers or its eradication. However, given the type of rearing characteristics (e.g., bordering transhumance) and animal trade (e.g., illegal markets in this region) this has not been possible. For example, Saudi Arabia serves as a major centre for international trade, where hundreds of thousands of small and large ruminants and camels are imported every year to the country for the purpose of slaughtering during the pilgrimage season. This trade in animals provides a significant risk of livestock disease transfer [16]. Additionally, in this region and its bordering countries transhumance rearing is very common, which leads to uncontrolled movements of animals within the country and across borders.

Other factors besides the economic level which drive the presence of SGP in the different reporting countries can be highlighted. For instance, reporting countries have other diseases which they prioritise over SGP. A study in India showed that most livestock producers do not perceive SGP to be a major problem compared to other common diseases. Additionally, less than half of the respondents of the survey were familiar with SGP [5]. In Morocco, a low-middle-income country, a series of outbreaks of Peste des Petits Ruminants (PPR) that occurred between 2007 and 2011 hindered the national vaccination campaigns for SGP [17], which consequently favoured the occurrence of sheep pox outbreaks, leading to a peak of outbreaks in 2010. Other countries, although they allocate economic resources to SGP vaccination, report that there is failure of vaccination which is likely to be caused by poor vaccine handling where the availability of electricity is limited for keeping the cold chain. Additionally, vaccination practice occurs when the outbreak is already in progress [18]. In Algeria, where sheep pox is still considered as a major animal health problem, despite a national control programme that continued for several decades, it has been hypothesised that the recurrence of SPPV is likely due to either internal spread within the country by transhumance animal movements which favour the contact of flocks with different immunisation status or to legal and illegal animal movements across uncontrolled borders or to a combination of both [19].

It is important to highlight that in this analysis, countries’ levels of endemicity were obtained according to the frequency of year they was reported during the study period. Thus, some countries have been classified here as endemic because they reported intermittently SGP throughout the 18-year study period, although they are officially considered free of the disease. Examples are Greece and Russia, that when presenting an SGP outbreak, promptly apply the corresponding control and eradication measures and therefore avoiding the disease to become endemic. They are, however, situated in a geographical position with bordering countries classified as highly endemic which enables the sporadic incursion of the disease into their territory.

Indeed, Bulgaria’s outbreaks reported in 2013 were in the same areas as the outbreak in 1995–1996, at the border with Turkey, and two other outbreaks occurred at the border with Greece. The most likely source of infection was animal movements from Turkey [4] and the illegal immigration of people. Similarly, Greece, which according to the criteria of this study is classified as endemic, has been reporting outbreaks since 2013, occurring in regions bordering Turkey. It is believed that sheep pox is introduced into Greece mainly from neighbouring countries to the east and is associated with the movements of infected sheep flocks close to the border and contacts between humans and animals and thus illegal immigrants and visitors could act as mechanical carriers [11]. In 2023, Spain reported its first outbreak in 50 years [12]. The source of the virus was never conclusively identified, though it has been connected to strains from North African origin, where the disease is endemic, possibly being introduced accidently through people from these countries working in sheep and goat farms in Spain [20]. Thus, European Member State countries face sporadic outbreaks due to accidental introduction via illegal immigration of humans and/or legal and illegal trade in animals across borders [19] from neighbouring endemic countries.

Likewise, Russia was free of the disease until 1993 when it reported an outbreak in a region bordering China [21], after which it has presented years free of the disease with outbreaks being reported intermittently during the years until 2008. Since then, it has reported low number outbreaks nearly every year (only the years 2009, 2014, and 2017 had no reported outbreaks in WAHIS, which made its classification in the present study as ‘endemic’). Furthermore, in 2020, unprecedented incursions of sheep pox outbreaks were reported in central Russia which were closely related to virulent circulating viruses from neighbouring regions [22]. Although Russia applies control measures (i.e., movement control, disease notification) and vaccination against SGP is practised, given its border areas of Kazakhstan, China, Mongolia, and the Caucasus countries, it may be almost impossible for Russia to become free of capripoxvirus disease in the near future [21].

As for the number of sheep and goat population as an influence on SGP emergence, countries with a high population of both sheep and goats were associated with higher endemicity and had a lower median GDP per capita. Regional differences in the animal populations were also observed. Indeed, East Asia and Central Asia had the highest number of sheep, and East Asia and Sub-Saharan Africa had the highest number of goats. These regions are represented by countries in the low-middle-income category and with the highest numbers of small ruminant populations. For example, Ethiopia, Chad, and Nigeria are the countries in Africa with the highest census of small ruminant population, with these being China and India for the Asian continent. In many of these countries, small ruminants are reared typically in either extensive transhumance (pastoral) or sedentary (back systems) and are generally kept for subsistence, rather than commercial purposes [23], which hinders the efforts to decrease the incidence of SGP. In the European region, Greece is the country of the European Union with the highest goat population and third highest country with the greatest number of sheep population [13] and with the most reported number of outbreaks. However, for the case of Greece, the population number of sheep and goats is most likely not an important factor. As previously mentioned, its reported outbreaks are due to its border with Turkey, a country classified here as highly endemic (and officially endemic to the disease), as the number of cases are low in relation with the number of outbreaks (i.e., the outbreaks are contained and thus the disease does not spread to the susceptible populations, which reduces the number of cases).

The multivariable analysis evaluating the risk of presenting an outbreak using the four variables, reflects the results of the description of the countries. Indeed, the income classification of a country was not found significant in the final multivariable model for the risk of presenting outbreaks. As previously mentioned, some upper level and high-income countries particularly those of the Middle East region still report high number of outbreaks and middle high-income countries which neighbour endemic countries present intermittent outbreaks. This is also shown when observing the region categories. The Middle East region had the highest incidence risk ratio (IRR) of presenting an outbreak (reference category Europe). This is followed by North Africa, which contrarily is represented mostly by countries in the lower-middle-income category. These two regions (Middle East and North Africa) are classified as a single region by FAO as the MENA region. It comprises 14 countries, extending from Iran in the East to Morocco in the West. This region has it particularities. On one hand, many countries, especially around the Mediterranean Sea, are highly dependent on agriculture, which plays an important role in their economies. On the other hand, about half of the countries are net oil exporters, with a weak agricultural sector [24]. Additionally, the in MENA region, sheep and goat husbandry is predominantly pastoral, with seasonal migration [23]. These aforementioned characteristics of the MENA region contribute to the higher risk of presenting a SGP outbreak.

The number of both sheep and goat population was also found significant in the multivariable model. As previously described, countries with high numbers of sheep and goats were all classified as endemic and thus the higher their numbers, the higher the IRR of presenting an outbreak. This is to be expected due to the contagious nature of the disease. SPPV and GTPV are highly contagious and spread through aerosols and/or close contact with infected animals and by indirect means such as contamination of cuts and abrasions [1]. This is in agreement with a study which reported that the seroprevalence of sheep and goat pox infection was significantly affected by herd size and crowding of animals that could facilitate the frequency of direct contact and hence accelerating the likelihoods of transmission [25]. It is also reported that in some countries in where the disease is endemic, in provinces where density of sheep is high and the grazing areas of different villages tend to overlap, the disease spreads quickly from one village to another [26].

The incidence rate for the category high number of goats was higher than the incidence rate of the category high number of sheep. This could be due to the fact that in some countries, the goat population is much higher than the sheep. For example, Nigeria has double the population of goats than sheep; in Oman, which reports high numbers of outbreaks, 80% of its small ruminant population are goats and Vietnam who has reported outbreaks of SGP, has zero sheep [13]. This highlights the importance of the role of goats in the epidemiology of SGP. It is generally considered that most strains of SPPV or GTPV have a host preference for either sheep or goats, but some strains of SPPV or GTPV could infect both sheep and goats [27,28]. Mixed reports are given on the role of goats in the epidemics of SGP. Some countries only report SPPV as responsible of the outbreaks, e.g., Morocco [8,9]. When analysing the epidemiology of sheep pox in Greece from 1987 to 2007, authors concluded that it was a SPPV rather than GTPV causing the outbreaks in sheep [11]. However, positive serology was present in goats with absence of clinical signs in mixed flocks with both species, with authors concluding that the involvement of goats in the epidemiology of outbreaks was unlikely [11]. This is in contrast to other reporting countries. In other sheep pox outbreaks in India, both species were affected by SPPV, with goats presenting mild clinical disease [28]. An outbreak of sheep pox in China was associated with GTPV in 2009 [29], in which only sheep where affected. A study in Ethiopia revealed that GTPV was the solely responsible for all investigated outbreaks in both sheep and goats throughout the study [30]. Iran reported a virulent goat pox infection in the ovine populations [31]. In experimental infection with either the Vietnam or Yemen isolate, goats developed more severe disease than sheep [32]. These findings suggest that goats play an important role in the epidemiology of SGP, even when it is only observed in sheep. To understand how the GTPV isolates may or not be involved in SGP epidemic, further investigation needs to be undertaken.

The descriptive situation of the reporting countries and the multivariable model show that there are major factors that influence the emergence or increase of number of outbreaks of SGP. These are as follows:

- *Characteristics of the agent*

Due to the very close antigenic relationship among *Capripoxviruses*, strains of SPPV and GTPV cannot be differentiated serologically [33]. Given the host preferences of SPPV or GTPV, the classification of *Capripoxvirus* is based on the animal species from which the virus is isolated, although different strains can affect both species [2]. Thus, strain identification based on the host species is not valid and it should be based on molecular diagnosis [6,8]. This however does not generally occur, particularly in low-income countries, and in livestock-producing countries with small herds and/or mixed herds, diagnosis is made by clinical signs and the species affected [9]. Additionally, a lack of trained veterinary staff to detected an outbreak in the field as early as possible and a lack of proper laboratory infrastructure hinders the ability to establish if the origin of the outbreak was due to an infected sheep or goat or both.

- *Vaccine and vaccination strategies*

All commercially available vaccines for SGP are live attenuated vaccines, prepared from a limited number of strains and currently, and there are no vaccines with a Differentiation of Infected from Vaccinated Animals (DIVA) component that are commercially available against SGP. None of these available vaccines are licenced within the EU and their use would inflict immediate restriction on the international trade of live sheep and goats [4]. A SGP DIVA vaccine would allow non-endemic countries to implement it for controlling outbreaks and would also be a useful tool for endemic countries to eventually acquire disease-free status following the implementation of an effective eradication campaign [3]. In spite of this limitation, vaccination is considered the only economically feasible method of controlling SGP outbreaks, decrease and contain its spread, and subsequently avoid disease-associated costs in countries where the disease is endemic [2,6]. However, even with the available effective vaccine, endemic countries have not been able to decrease the number of outbreaks to a minimum number, eradicate, nor be free of the disease for more than a certain amount years. This is because the ability of the vaccines to provide good protection and control the outbreaks is dependent on the maintenance of sufficient herd immunity (over 80%) by carrying out annual vaccination [34]. Many endemic countries do not have a well-organised vaccination programme and it is practised only when suspected outbreaks are reported to the veterinary authority, which is then notified [1]. This lack of compulsory and consistent vaccination strategies lead to the required herd immunity not being reached. A study from a state in India reported only a 2.22% vaccination coverage on its sheep population [27], with this possibly being one of the major factors contributing to disease outbreaks. Morocco, on the other hand, which had a high level of vaccination coverage, reaching 80% during the 1996 to 2006 decade, reported a decline of outbreaks [9,17]. However, after the declaration of the Peste des petits ruminants in Morocco, the fight against sheep pox was focused on the management of reported outbreaks, leading to a decrease of vaccination, causing the resurgence of a high number of outbreaks.

- *Livestock production systems*

The husbandry methods prevalent in certain countries predispose to the rapid spread of Capripox virus. Indeed, these viral infections occur predominantly in countries with extensive, nomadic or transhumance breeding of sheep and goats, and mainly in regions where uncontrolled movements of live animal are prevalent. In the North African, Middle East region and Indian subcontinent where SGP is endemic for example the highland mixed farming system is the most important and prevalent. This type of system livestock is based primarily on the raising of livestock on communally managed land. In some cases, both the livestock and the people who control them are transhumant, migrating seasonally [23]. In transhumant pastoral communities there is periodic uncontrolled influx of ruminant flocks from neighbouring countries as well as inter district animal movement in particular in the dry season [34,35]. These conditions make reporting as well is passive, with little if any active disease surveillance [5] and the uncontrolled movement of animals increases the introduction of SGP.

- *Political instability*

Conflict in countries leads to collapsed veterinary services and a lack of available vaccines and medicines, leading to delayed or failed containment of epidemics of many veterinary and human infectious disease. Additionally, it promotes the mass movement of refugees which may also travel with unvaccinated cattle, sheep, and goats. As an example, during the Syrian conflict, a 60% increase in the quantity of livestock near the Syrian border was reported [3]. Moreover, following the collapse of the Syrian veterinary services in 2012, uncontrolled livestock movements increased significantly. Turkey is the only neighbouring country with a relatively strict border control system, requiring the slaughter of all non-registered animals. Thus, unvaccinated live animals were being legally imported, or illegally crossing into Iraq, Jordan, and Lebanon, sometimes without quarantine, for sale on the open market and slaughterhouses throughout those countries [4]. All these factors drive the increase of SGP outbreaks (and other livestock diseases) in the affected country and given that vaccination of cattle and small ruminants in the war-torn areas is neither possible nor safe to perform, it is highly likely that the conflict regions will continue to serve as a source of infection until reconstruction of basic infrastructure can be commenced. Furthermore, political instability makes the surveillance and obtaining data nearly impossible, with countries having only sparse data on diseases.

The outbreak analysis shows that low-middle-income countries are those with the greatest number of outbreaks and the top six countries with number of outbreaks are also those countries with the highest number of sheep and goat populations. When observing outbreaks evolution throughout the 18-year period, overall, their total numbers have been decreasing. Africa and Asia differed in the peaks but had a similar total number of outbreaks overall through the period. Europe had the lowest number of outbreaks, with some years recording zero number of outbreaks. The results show that the evolution of the disease in the study period is influenced by the same countries of the regions: North African countries, countries in the Middle East, and East Asian countries—China and India in particular. Regionally, for Africa, North Africa had higher number of outbreaks during all the years, even though this region is represented by a smaller number of countries than the Sub-Saharan region. For the Asian continent, the Middle East presented increases and decreases of outbreaks that were the same as Central Asia. East Asia, although it had the highest number of outbreaks, steadily decreased its number of outbreaks.

These same countries that report the highest number of outbreaks are those who influence the peaks in a given year and once they control the epidemic, there is a decrease in the total which produce the change point. Indeed, the year 2006 of the first segment had the second highest total number of outbreaks for the whole study period because India reported 58.76% of the total outbreaks. The first change point corresponds to the situation of the disease in 2009–2010, in which outbreaks were increasing in the African continent, in particular in Morocco, which in 2010 experienced sheep pox epidemic [1], recording the second segment with the highest number outbreaks for the whole study period. By controlling the epidemic, the second change point occurs as there is a sharp decline immediately in the year 2011, with a decrease of number of outbreaks in the African continent until 2014. In 2014, Africa had a decline in number of outbreaks; indeed, all six top-reporting nations of this continent reported the lowest number of outbreaks of the study period (excluding the year 2005), especially Morocco. In this same year, Asia showed a rising number of outbreaks, which is shown in the third change point. This reached its peak in 2015, which was attributed to the rise of outbreaks in Oman and Iran not decreasing its number of outbreaks in the previous year, and the African countries Niger (which in 2014 had very low number of outbreaks) and Tunisia contributing, with the highest number of outbreaks. The fourth change point and last segments are given by the lowest number of outbreaks recorded in the last years of the study period. This could be attributed to the fact that for the last three years of the study, many countries did not submit reports (Table A1), including countries such as China which was one of the top six countries with the greatest number of outbreaks during the 18-year study period. Thus, the lower number of outbreaks could be caused by lack of data and not due to countries having SGP under control. Indeed, even with the higher number of no-submission of reports in the last 3 years of the study period, it is important to highlight that the year 2021 reported a slight increase of reported outbreaks for both Africa and Asia mostly contributed by Algeria, Tunisia, Morocco, Iran, and Turkey.

The results present a general picture of the evolution and factors of SGP emergence. However, there are some limitations to this study, and indeed these results are to be interpreted with caution. First, the outcome variable used in the analysis is the number of outbreaks of SPG, and SGP being is a neglected, under-reported disease. Thus, many countries lacked proper quantitative data and there were many missing reports, which added to missing values to the data. Secondly, the classification of SGP status in a country was given by the frequency of reporting SGP; therefore, here they are classified as ‘endemic’, but officially they are free of the disease. The last point to be taken into account is that here, sheep pox and goat pox diseases are treated as a single disease, as reported to the WOAH. Therefore, from the data available it is not possible to determine which virus (SPPV or GTPV) was causing each outbreak.

## 5. Conclusions

Based on the results presented here, the presence of SGP is conditioned by the circumstances of the affected countries. Countries such as EU Member States, who only present the disease sporadically, have more intensive farming which enables better biosecurity measures, have higher laboratory capabilities, and EU legislation to be followed in the case of a SGP outbreak. These measures include stamping out the virus by killing animals, safe disposal of carcasses and risk products, official cleaning and disinfection, and the implementation of restricted, protection, and surveillance zones. This is in contrast with the endemic countries that do not have or cannot implement such measures, thus conditioning the spread to other countries.

Indeed, when observing the outbreak trend over the study period years, it is noticeable that it is the same affected countries which contribute to the increase or decrease of outbreaks in a particular period of time. Thus, the control of SGP in endemic countries would bring an overall decrease of SGP. Although different constraints in endemic countries make eradication highly unlikely, progressive control and even regional elimination of infection could be feasible. This would be possible by giving priority to the disease by the national veterinary services of affected countries and through well-designed and shared control strategies between neighbouring and affected countries. A great example was the RADISCON (Regional Animal Disease Surveillance and Control Network) programme which was created in 1996 for a period of 5 years [36], which supported/promoted the implementation health measures to control infectious diseases that had a serious economic impact on the affected country. The implementation of this programme greatly improved the animal health status in Morocco, Algeria, Tunisia, and Libya, which witnessed a major decrease in outbreaks of sheep pox [17]. Key points that need to be implemented for the control of SGP are the implementation of mass vaccination programmes, mainly in low-income countries where the livestock capital is jeopardised because of the disease (higher income countries like EU ones will prefer not to vaccinate—at least with the currently available vaccine—and have the capacity to control SGP by zoning, testing and culling), the need for a strategy to regulate transhumance and uncontrolled movement of animals, and the addition of goats as a target species in the vaccination programmes that are performed, as goats may or may not play a significant role in spreading the virus in affected countries. This would bring economic benefits and improve the status of animal health.

## Figures and Tables

**Table 1:** Total number of reports submitted to the WOAH by each country (2005–2022). Data are organised by continents and regions.

Number of Reports	Africa		Asia			Europe	Total *n* of Countries
	North Africa	Sub-Saharan Africa	Middle East	Central Asia	East Asia		
18	Algeria, Egypt, Morocco, Tunisia	Rep. of the Congo, Ethiopia, Kenya, Lesotho, Nigeria, Somalia, Sudan, Uganda	Bahrain, Iraq, Israel, Kuwait, Palestine, United Arab Emirates, Qatar, Saudi Arabia	Afghanistan, Azerbaijan, Kyrgyzstan, Pakistan, Republic of Türkiye	Bhutan, Taiwan, India, Laos, Mongolia, Nepal, Sri Lanka, Indonesia	Russia, Spain	35
17		Tanzania, Chad, Cote d’ Ivoire, Eritrea, Níger, Zambia	Jordan, Oman, Lebanon	Iran, Turkmenistan	Rep. of Korea, Vietnam		13
16		Togo		Kazakhstan		Bulgaria	3
15	Libya	Ghana, Mali, Mauritania, Senegal, Burkina Faso			People’s Rep. of China		7
14			Yemen	Tajikistan	Bangladesh	Greece	4
13		Cameroon					1
12		Rwanda					
11		Burundi					1
10		Sierra Leone					1
5		Gambia					1
Total	5	25	12	9	12	4	67

**Table 2:** Results of univariate logistic regression, number of sheep, goats, income category of reporting country (*n* = 53).

Variable	Category	OR (95% CI)	*p*-Value
Number of sheep	Low	Ref	–
	Middle low	6.76 (4.27–10.70)	<0.001
	Middle high	2.96 (2.01–4.36)	<0.001
	High	7.66 (4.77–12.30)	<0.001
Number of goats	Low	Ref	–
	Middle low	4.03 (2.71–5.99)	<0.001
	Middle high	13.97 (8.11–24.06)	<0.001
	High	6.79 (4.37–10.53)	<0.001
Income category	Low	Ref	–
	Lower middle	0.26 (0.15–0.44)	<0.001
	Upper middle	0.2 (0.11–0.36)	<0.001
	High	0.3 (0.16–0.55)	<0.001

Legend: OR—odds ratio; CI—confidence interval; Ref.—reference category.

**Table 3:** Number of outbreaks, countries (*n =* 53), and regions by SGP status classification and income category.

	Country Income Category											
	Low Income			Lower Middle Income			Upper Middle Income			High Income		
Numbers	Outbreaks	Countries/Regions		Outbreaks	Countries/ Regions		Outbreaks	Countries/ Regions		Outbreaks	Countries/ Regions	
SGP status classification												
Highly endemic	7621	8	CA = 1 SS = 6 ME = 1	18,805	8	CA = 2 NA = 3 EA = 1 SS = 1 ME = 1	5725	4	CA = 1 EA = 1 ME = 1 NA = 1	3840	2	ME = 2
Endemic	16	2	SS = 2	488	10	CA = 2 NA = 1 EA = 3 SS = 4	92	2	CA = 1 EU = 1	884	4	EU = 1 ME = 3
Low endemic	2	1	SS = 1	98	5	EA = 2 SS = 3	1	1	CA = 1			
Sporadic				3	2	SS = 2	5	2	CA = 1 EU = 1	24	2	EA = 1 EU = 1
Total	7639	11		19,394	25		5825	9		4748	8	

Legend: NA—North Africa; SS—Sub-Saharan; ME—Middle East; CA—Central Asia; EA—East Asia; EU—Europe.

**Table 4:** Results of the multivariable negative binomial model for the risk of presenting an outbreak associated with number of sheep, goats, and region.

Explanatory Variable	Category	IRR (95% CI)	*p*-Value
Number of sheep	Low	Ref	–
	Middle low	1.82 (0.95–3.48)	0.071
	Middle high	4.55 (1.96–10.58)	<0.001
	High	9.87 (3.89–25.03)	<0.001
Number of goats	Low	Ref	–
	Middle low	2.05 (1.22–3.44)	0.007
	Middle high	6.03 (2.80–12.98)	<0.001
	High	13.85 (5.43–35.29)	<0.001
Region	Europe	Ref	–
	Central Asia	2.26 (0.95–5.38)	0.065
	East Asia	2.88 (1.12–7.45)	0.029
	Middle East	76.63 (30.74–191.01)	<0.001
	North Africa	56.25 (21.60–146.54)	<0.001
	Sub-Saharan Africa	1.54 (0.65–3.68)	0.328

Legend: IRR—incidence risk ration; CI—confidence intervals; Ref.—reference category.

## Data Availability

The data that support the findings of this study are available from the corresponding author upon reasonable request.

## Abbreviations

The following abbreviations are used in this manuscript:

GDP	Gross Domestic Product
GTP	Goat pox
SPP	Sheep pox
SPPV	Sheeppox virus
SGP	Sheep and goat pox
GTPV	Goatpox virus
LSDV	Lumpy Skin Disease Virus
WAHIS	World Animal Health Information System
WOAH	World Organisation for Animal Health

## Appendix A

**Table A1:** Classification of sheep- and goat-pox-reporting countries by region and income group classification. Number of reports submitted and total number of outbreaks and cases by country.

Region		Country	Number of Reports	*n* of Times Reported	% of Number of Time	Outbreaks Total	Cases Total	Year Where Reports Is Missing	Income Group Classification **
Africa									
	North Africa	Algeria	18	17	94.4%	1720	15,546		LMI
		Egypt	18	3	16.7%	7	20		LMI
		Libya	15	7	46.7%	67	38,242	2020, 2021, 2022	UMI
		Morocco	18	18	100%	4766	30,726		LMI
		Tunisia	16	16	100%	2555	13,487	2021, 2022	LMI
	Sub-Saharan	Burkina Faso	15	6	40%	31	1628	2020, 2021, 2022	LI
		Burundi	11	1	9.1%	5	220	2011, 2013, 2014, 2019, 2020, 2021, 2022	
		Cameroon	13	6	46.2%	49	1147	2018, 2019, 2020, 2021, 2022	LMI
		Chad	17	2	11.8%	2	37	2022	LI
		Congo Rep. of the	17	1	5.9%	2	229		LMI
		Cote D’Ivoire	17	2	11.8%	2	445	2021	LMI
		Eritrea	17	17	100%	85	36,196	2022	LI
		Ethiopia	18	18	100%	3371	269,802		LI
		Gambia	5	1	20%	7	16	2005–2007, 2010–2015, 2019–2022	
		Ghana	15	4	26.7%	26	32	2020, 2021, 2022	LMI
		Kenya	18	4	22.2%	5	18		LMI
		Lesotho	18	1	5.6%	1	25		LMI
		Mali	15	7	46.7%	11	1531	2020, 2021, 2022	LI
		Mauritania	15	1	6.7%	8	113	2020, 2021, 2022	LMI
		Niger	16	11	68.8%	2445	31,075	2020, 2021, 2022	LI
		Nigeria	18	6	33.3%	117	1498	2022	LMI
		Rwanda *	12	6					
		Senegal	15	14	93.3%	112	2993	2020, 2021, 2022	LMI
		Sierra Leone	10	0	0%	0	458	2005–2008, 2019–2022	
		Somalia	18	11	61.1%	568	21,348		LI
		Sudan	18	18	100%	296	19,694		LI
		Tanzania	15	4	26.7%	25	600	2020, 2021, 2022	LMI
		Togo *	16	1					
		Uganda	18	0	0%	0	78		LI
		Zambia *	17	1					
Asia									
	Central Asia	Afghanistan	18	10	55.6%	284	12,064		LI
		Azerbaijan	18	1	5.6%	1	1		UMI
		Iran	17	17	100%	4777	71,344	2022	LMI
		Kazakhstan	16	5	31.3%	8	515	2020, 2021	UMI
		Kyrgyzstan	17	8	47.1%	24	226	2022	LMI
		Pakistan	18	5	27.8%	33	64,444		LMI
		Tajikistan	15	9	60%	33	270	2020, 2021, 2022	LMI
		Turkey	18	18	100%	2137	87,718		UMI
		Turkmenistan	17	1	5.9%	1	245	2005	UMI
	East Asia	Bhutan	18	2	11.1%	3	29		LMI
		Bangladesh *	14	12					
		China	15	15	100%	1949	68,520	2020, 2021, 2022	UMI
		Chinese Taipei	18	4	22.2%	583	7289		
		India	18	18	100%	3938	91,618		LMI
		Indonesia *	18	5					
		Korea Rep. of	16	1	6.3%	1	1	2021, 2022	HI
		Laos *	18	3					
		Mongolia	16	8	50%	161	36,146	2020, 2021	LMI
		Nepal	18	6	33.3%	48	5441		LMI
		Sri Lanka	18	1	5.6%	1	1186		LMI
		Vietnam	17	2	11.8%	16	4300	2022	LMI
	Middle East	Bahrain	18	10	55.6%	601	3460		HI
		Iraq	18	17	94.4%	1561	24,341		UMI
		Israel	18	13	72.2%	77	2026		HI
		Jordan	17	17	100%	129	1907	2021	LMI
		Kuwait	18	9	50%	106	1967		HI
		Lebanon *	17	7					
		Qatar *	18	3					
		Oman	17	14	82.4%	3538	42,461	2022	HI
		Palestine	18	18	100%	989	8978		
		Saudi Arabia *	18	7					
		United Arab Emirates	18	4	22.2%	27	1915		HI
		Yemen	18	9	50%	442	13,290	2022	LI
Europe									
	Europe	Bulgaria	16	1	6.3%	4	64	2021, 2022	UMI
		Greece	15	8	53.3%	150	2120	2020, 2021, 2022	HI
		Russia	18	12	66.7%	86	4176		UMI
		Spain	18	1	5.6%	23	464		HI

Legend: * Only reported the disease as present or suspicion, with no quantitative data; ** Income group classification by the World Bank 2023. LI—low income; LMI—lower middle income; UMI—upper middle income; HI—high income.

**Table A2:** Results of rank sum test comparing the median number of sheep of each region.

Sheep	Region				
	East Asia	Europe	Middle East	North Africa	Sub-Saharan Africa
Central Asia	*p* < 0.0033		*p* < 0.0033		*p* < 0.0033
East Asia		*p* < 0.0033			*p* < 0.0033
Europe			*p* < 0.0033	*p* < 0.0033	*p* < 0.0033
Middle East					
North Africa					

Legend: Bonferroni-adjusted *p*-value significant at <0.0033.

**Table A3:** Results of rank sum test comparing the median number of goats of each region.

Goats	Region				
	East Asia	Europe	Middle East	North Africa	Sub-Saharan Africa
Central Asia		*p* < 0.0033	*p* < 0.0033		*p* < 0.0033
East Asia			*p* < 0.0033		
Europe			*p* < 0.0033	*p* < 0.0033	*p* < 0.0033
Middle East				*p* < 0.0033	*p* < 0.0033
North Africa					*p* < 0.0033

Legend: Bonferroni adjusted *p*-value significant at <0.0033.

## References

[1] World Organisation for Animal Health (2024). Terrestrial Animal Health Code.

[2] Babiuk S., Bowden T.R., Boyle D.B., Wallace D.B., Kitching R.P. (2008). Capripoxviruses: An emerging worldwide threat to sheep, goats and cattle. Transbound. Emerg. Dis..

[3] Tuppurainen E.S.M., Venter E.H., Shisler J.L., Gari G., Mekonnen G.A., Juleff N., Lyons N.A., De Clercq K., Upton C., Bowden T.R. (2017). Review: Capripoxvirus Diseases: Current Status and Opportunities for Control. Transbound. Emerg. Dis..

[4] Garner M.G., Sawarkar S.D., Brett E.K., Edwards J.R., Kulkarni V.B., Boyle D.B., Singh S.N. (2000). The extent and impact of sheep pox and goat pox in the state of Maharashtra, India. Trop. Anim. Health Prod..

[5] Hamdi J., Munyanduki H., Omari Tadlaoui K., El Harrak M., Fassi Fihri O. (2021). Capripoxvirus Infections in Ruminants: A Review. Microorganisms.

[6] Bhanuprakash V., Indrani B.K., Hosamani M., Singh R.K. (2006). The current status of sheep pox disease. Comp. Immunol. Microbiol. Infect. Dis..

[7] Zro K., Zakham F., Melloul M., El Fahime E., Ennaji M.M. (2014). A sheeppox outbreak in Morocco: Isolation and identification of virus responsible for the new clinical form of disease. BMC Vet. Res..

[8] Lafar S., Zro K., Haegeman A., Khayli M., Clercq K.D., Lancelot R., Ennaji M.M. (2019). Clinical and Epidemiological Evolution of Sheep Pox in Morocco. J. Agric. Sci. Technol. A.

[9] Garner M.G., Lack M.B. (1995). Modelling the potential impact of exotic diseases on regional Australia. Aust. Vet. J..

[10] Mangana O., Kottaridi C., Nomikou K. (2008). The epidemiology of sheep pox in Greece from 1987 to 2007. Rev. Sci. Tech. (Int. Off. Epizoot.).

[11] (2024). WAHIS: World Animal Health Information System. https://wahis.woah.org/#/home.

[12] FAO (2023). Food and Agriculture Organization of the United Nations. https://www.fao.org/faostat/en/#data/QCL.

[13] (2017). The World Bank (WB). https://data.worldbank.org/indicator/NY.GDP.PCAP.KD.

[14] Aminikhanghahi S., Cook D.J. (2017). A Survey of Methods for Time Series Change Point Detection. Knowl. Inf. Syst..

[15] EFSA European Food Safety Authority (2014). Scientific Opinion on sheep and goat pox. EFSA J..

[16] Boshra H., Truong T., Babiuk S., Hemida M.G. (2015). Seroprevalence of Sheep and Goat Pox, Peste Des Petits Ruminants and Rift Valley Fever in Saudi Arabia. PLoS ONE.

[17] Ben Chehida F., Ayari-Fakhfakh E., Caufour P., Amdouni J., Nasr J., Messaoudi L., Haj Ammar H., Sghaier S., Bernard C., Ghram A. (2018). Sheep pox in Tunisia: Current status and perspectives. Transbound. Emerg. Dis..

[18] Tadesse B., Aregahagn S., Muluneh B.T., Worku Y. (2024). Spatio-temporal ditribution and transmission dynamics of sheep pox and goat pox diseases in South Wollo zone north East Ethiopia. Heliyon.

[19] Achour H.A., Bouguedour R. (1999). Epidemiology of sheep pox in Algeria. Rev. Sci. Tech. (Int. Off. Epizoot.).

[20] Maksyutov R.A., Gavrilova E.V., Agafonov A.P., Taranov O.S., Glotov A.G., Miheev V.N., Shchelkunov S.N., Sergeev A.N. (2015). An Outbreak of Sheep Pox in Zabajkalskij kray of Russia. Transbound. Emerg. Dis..

[21] Krotova A., Shalina K., Mazloum A., Kwon D., Van Schalkwyk A., Byadovskaya O., Sprygin A. (2022). Genetic characterization of sheep pox virus strains from outbreaks in Central Russia in 2018–2019. Transbound. Emerg. Dis..

[22] Dixon J., Gulliver A., Gibbon D. (2001). Farming Systems and Poverty: Improving Farmers’ Livelihoods in a Changing World.

[23] Mahmah A., Amar A. (2021). Food Security in the MENA Region: Does Agriculture Performance Matter?. Emerging Challenges to Food Production and Security in Asia, Middle East, and Africa, Climate Risks and Resource Scarcity.

[24] Dubie T., Dagnew B., Hamid M., Bizuayehu F., Fentahun G. (2022). Seroprevalence and associated risk factors of pox infection among sheep and goats in selected districts of Afar region, Ethiopia. Heliyon.

[25] Kitching R.P., McGrane J.J., Taylor W.P. (1986). Capripox in the Yemen Arab Republic and the Sultanate of Oman. Trop. Anim. Health Prod..

[26] Bhanuprakash V., Moorthy A.R., Krishnappa G., Srinivasa Gowda R.N., Indrani B.K. (2005). An epidemiological study of sheep pox infection in Karnataka State, India. Rev. Sci. Tech. (Int. Off. Epizoot.).

[27] Bhanuprakash V., Venkatesan G., Balamurugan V., Hosamani M., Yogisharadhya R., Chauhan R.S., Pande A., Mondal B., Singh R.K. (2010). Pox outbreaks in sheep and goats at Makhdoom (Uttar Pradesh), India: Evidence of sheeppox virus infection in goats. Transbound. Emerg. Dis..

[28] Yan X.M., Chu Y.F., Wu G.H., Zhao Z.X., Li J., Zhu H.X., Zhang Q. (2012). An outbreak of sheep pox associated with goat poxvirus in Gansu province of China. Vet. Microbiol..

[29] Gelaye E., Belay A., Ayelet G., Jenberie S., Yami M., Loitsch A., Tuppurainen E., Grabherr R., Diallo A., Lamien C.E. (2015). Capripox disease in Ethiopia: Genetic differences between field isolates and vaccine strain, and implications for vaccination failure. Antivir. Res..

[30] Alwan H., Torabi M., Nourani H., Al-Shuhaib M.B.S. (2023). The emergence of novel Iranian variants in sheeppox and goatpox viral envelope proteins with remarkably altered putative binding affinities with the host receptor. Virus Genes.

[31] Babiuk S., Bowden T.R., Parkyn G., Dalman B., Hoa D.M., Long N.T., Vu P.P., Bieu do X., Copps J., Boyle D.B. (2009). Yemen and Vietnam capripoxviruses demonstrate a distinct host preference for goats compared with sheep. J. Gen. Virol..

[32] Balinsky C.A., Delhon G., Smoliga G., Prarat M., French R.A., Geary S.J., Rock D.L., Rodriguez L.L. (2008). Rapid preclinical detection of sheeppox virus by a real-time PCR assay. J. Clin. Microbiol..

[33] Kardjadj M. (2017). Prevalence, distribution, and risk factor for sheep pox and goat pox (SPGP) in Algeria. Trop. Anim. Health Prod..

[34] Nizeyimana G., Vudriko P., Erume J., Mubiru F., Eneku W., Biryomumaisho S., Mwebe R., Arinaitwe E., Ademun R., Atim S. (2023). Spatio-temporal analysis of sheep and goat pox outbreaks in Uganda during 2011–2022. BMC Vet. Res..

[35] FAO (2024). RADISCON Phase Two Project Presented to Donors. Food and Agriculture Organization of the United Nations. https://www.fao.org/4/y3428e/y3428e08.htm.

[36] Cáceres G.G., Romero G.L., Bonilla G.S., Guerrero C.F., Fernandez M.M., Capilla G.J., Tejero C.J. (2024). Description of sheep pox outbreak in Spain in 2022–2023: Challenges found and lessons learnt in relation with control and eradication of this disease. Viruses.

